# Supplementary material for: Promotion of tumor angiogenesis and growth induced by low-dose antineoplastic agents via bone-marrow-derived cells in tumor tissues
Source: Front Pharmacol. 2024 Jul 25;15:1414832. doi: 10.3389/fphar.2024.1414832 (PMC11306047; doi:10.3389/fphar.2024.1414832)

Supplementary Figure 1

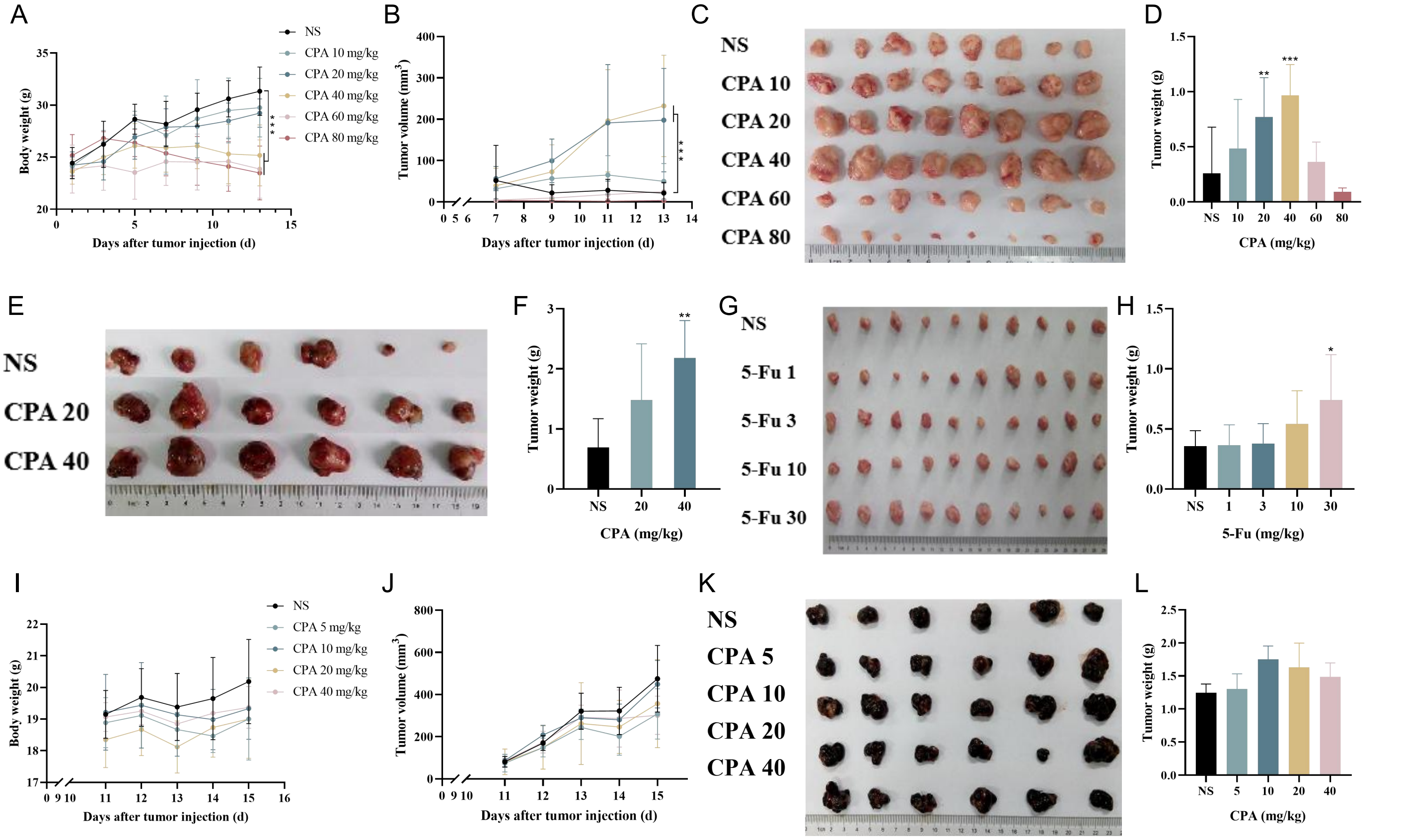

Supplementary Figure 1 Low-dose antineoplastic agents promoted tumor growth *in vivo*.
A-D, S180 tumor growth of mice treated with vehicle alone or the indicated dosages of CPA (n=8/group) A. Body weight; B. Tumor volume, monitored every two days; C. Macroscopic appearance of S180 tumors; D. Tumor weight; E-F, LLC tumor growth of mice treated with vehicle alone or the indicated dosages of CPA (n=6/group) E. Macroscopic appearance of LLC tumors; F. Tumor weight; G-H, S180 tumor growth of mice treated with vehicle alone or the indicated dosages of 5-Fu (n=10/group) G. Macroscopic appearance of S180 tumors; H. Tumor weight; I-L, Growing B16 tumor growth of mice treated with vehicle alone or the indicated dosages of CPA (n=6/group) I. Body weight; J. Tumor volume; K. Macroscopic appearance of B16 tumors; L. Tumor weight. *vs* control, *, $P < 0.05$; **, $P < 0.01$; ***, $P < 0.001$.

Supplementary Figure 2

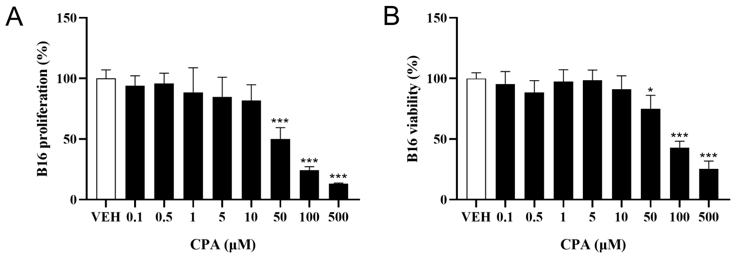

Supplementary Figure 2 Low-dose CPA did not show promoting effects in tumor cells proliferation and viability *in vitro*.
A. The proliferation of B16 treated with low dose of CPA (n=6/group); B. The viability of B16 treated with low dose of CPA (n=6/group). *vs* control, *, $P < 0.05$; ***, $P < 0.001$.

Supplementary Figure 3

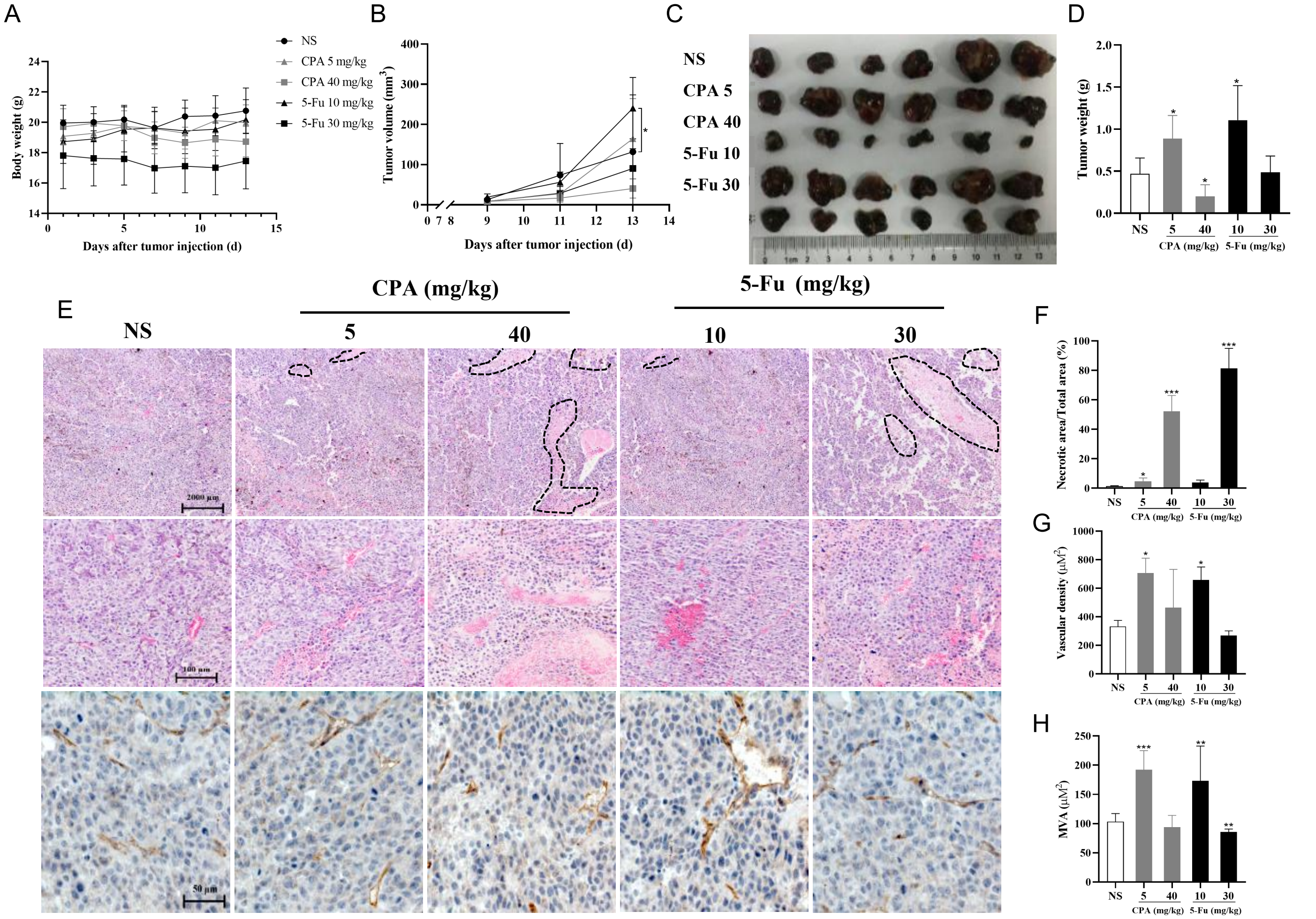

Supplementary Figure 3 Comparison of anti-tumor efficacy and micro-vessel area between high-dose and low-dose CPA and 5-Fu treated B16 tumor models.

A-D, B16 tumor growth of mice treated with vehicle alone (normal saline) or the indicated dosages of CPA and 5-Fu (n=6/group) A. Body weight; B. Tumor volume, monitored every two days; C. Macroscopic appearance of B16 tumors; D. Tumor weight; E. Tumor necrosis, tumor vessel and micro-vessel of B16 tumor tissues; F. Effects of CPA or 5-Fu on tumor necrosis of B16 tumor tissues; G. Effects of CPA or 5-Fu on vascular density of B16 tumor tissues; H. Effects of CPA or 5-Fu on micro-vessel area of B16 tumor tissues. *vs* control, *, $P < 0.05$; **, $P < 0.01$; ***, $P < 0.001$.

Supplementary Figure 4

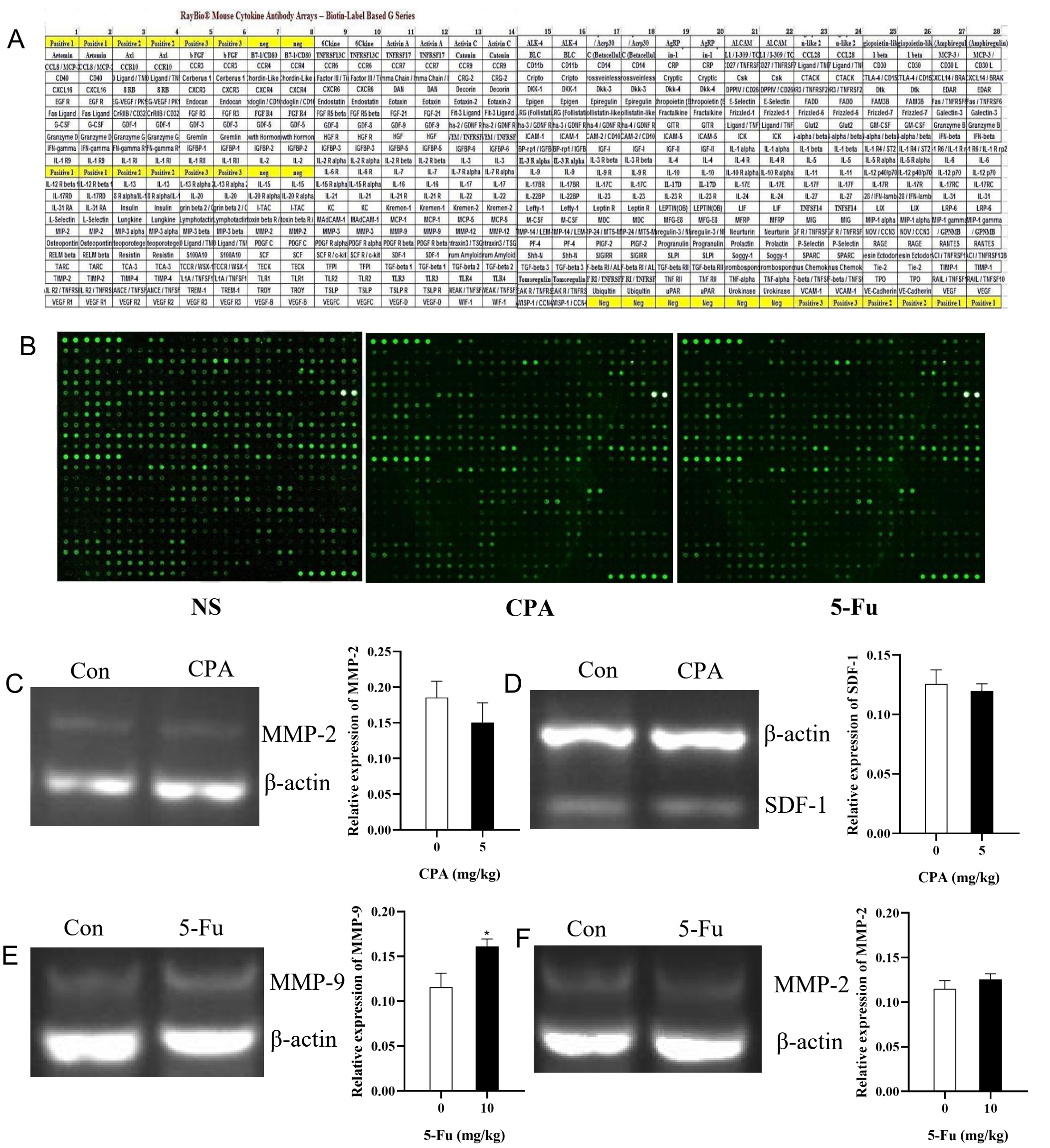

Supplementary Figure 4 Low-dose CPA and 5-Fu promoted recruitment of BMDCs to tumor tissues and expression of pro-angiogenic factors.
A. The protein chip assay; B. The protein expression patterns of B16 tumors from mice treated with vehicle alone, CPA and 5-Fu; C-D, Effects of low-dose CPA on mRNA transcriptions of MMP-2, SDF-1 in B16 tumor tissues (n=3/group); E-F, Effects of low-dose 5-Fu on mRNA transcriptions of MMP-9, MMP-2 in B16 tumor tissues (n=3/group). *vs* control, *, $P < 0.05$.

Table 1. Primer sequences for RT-PCR.

Primers	Sequences (5'→3')	Product size (bp)
Mouse SDF-1 (F)	AGCCAACGTCAAGCATCTG	106
Mouse SDF-1 (R)	TAATTTCGGGTCAATGCACA	
Mouse β-actin (F)	GAGACCTTCAACACCCCAGC	263
Mouse β-actin (R)	ATGTCACGCACGATTTCCC	
Mouse $VEGFR_2$ (F)	CGCTCAGTGATGTAGAGGAAG	343
Mouse $VEGFR_2$ (R)	CAGAGCAACACACCGAAAGAC	
Mouse MMP-2 (F)	TAGACCTCAGCTTGCCCATT	402
Mouse MMP-2 (R)	CCTTGGTGGAACAGAAGGAA	
Mouse MMP-9 (F)	GGCACCTACTTGCTCACCTC	106
Mouse MMP-9 (R)	GTGTGTGTGTATGCCCAAGC	

Affinibody marker (6.5-270 kDa)   AIWB-011

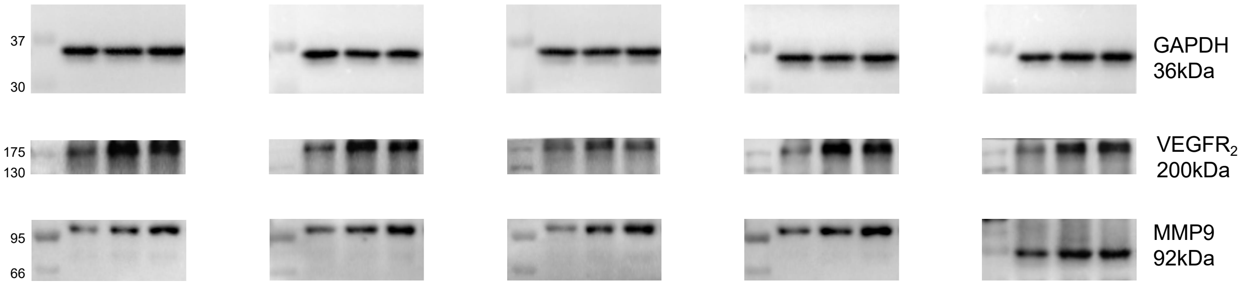

Supplement: Supplementary file 1 [file DataSheet1.PDF]
